# An empirical study of intermediate and advanced Chinese learners of Japanese language about reading strategies based on think-aloud protocols

**DOI:** 10.3389/fpsyg.2026.1773749

**Published:** 2026-05-29

**Authors:** Yang Sun, Min Zhang, Shang Tan

**Affiliations:** 1 School of Foreign Languages, Yangzhou University, Yangzhou, China; 2 School of Foreign Languages, Yangzhou Polytechnic University, Yangzhou, China; 3 Hangzhou Entel Foreign Language School, Hangzhou, China

**Keywords:** classroom instruction, intermediate and advanced Chinese learners, learners of Japanese language, reading strategies, think aloud protocols

## Abstract

**Introduction:**

Since the 1970s, significant progress has been made in the field of L2 education regarding reading strategies, both theoretically and practically. However, there is limited research specifically on reading strategies for Chinese learners of Japanese. As the most authoritative test, JLPT (Japanese-Language Proficiency Test) has been widely valued, and the number of applicants has increased year by year. Therefore, in order to provide reference for learners of Japanese language, we conducted an questionnaire on Japanese reading strategies based on think-aloud protocols (TAPs) with 28 valid participants.

**Methods:**

In this paper, 30 s and third grade college students were initially recruited, and 2 participants with prior familiarity with the experimental materials were excluded, with a final valid sample of 28 students selected by TAPs, and N1 test was used as experimental material. Before the experiment, several survey questions were drawn up. It can be summarized as follows: (1) What are the characteristics of reading strategies used by intermediate and advanced Chinese learners of Japanese language? (2) What is the relationship between the number of reading strategies used by intermediate and advanced learners and their reading scores? (3) What are the differences in reading strategies used by intermediate and advanced learners? Then, the learners’ think aloud data were textualized and the reading strategies used by individual learners were classified according to the SORS’ classification benchmarks for reading strategies. To further explore the relationship between reading strategies and reading scores, several SPSS statistical methods were also used to analyze the data in this study.

**Results:**

(1) There was a moderate positive correlation between the use of global reading strategies used by all learners and their reading scores (*r* = 0.61, *p* = 0.000). There was a moderate negative correlation between the use of problem-solving strategies and the reading scores of all learners (*r* = −0.47, *p* = 0.01). (2) Advanced learners used problem-solving strategies the least. There was a moderate positive correlation between global reading strategies and reading scores used by advanced learners (*r* = 0.48, *p* = 0.03). (3) Intermediate learners use global reading strategies the least. There was a moderate positive correlation between global reading strategies and reading scores used by intermediate learners (*r* = 0.48, *p* = 0.03). (4) There were significant differences among global reading strategies, problem-solving strategies and support reading strategies used by intermediate and advanced learners.

**Discussion:**

(1) A greater use of global reading strategies was associated with higher reading scores, while increased use of problem-solving strategies was associated with lower reading scores for all learners. (2) Advanced learners who used more global reading strategies had higher reading scores. (3) The more global reading strategies intermediate learners used, the higher their reading scores were. (4) Significant differences were observed in the reading strategies used by intermediate and advanced learners. According to the differences, some constructive suggestions can be put forward in Japanese reading teaching.

## 1 Introduction

For second language learners, reading is a crucial activity that directly impacts learning outcomes. Reading strategies include a variety of methods, techniques, and behaviors that learners use to achieve comprehension or address challenges. These strategies are widely recognized as the most effective means for enhancing reading proficiency (Baker and Brown, 1984). Thus, exploring reading strategies is crucial for improving learners’ reading proficiency and overall learning effectiveness. In the domain of second language reading strategies research, a considerable amount of research has been conducted in the field of second language reading strategies to examine which strategies are effective and how to apply them effectively (Baker and Brown, 1984; Hong-Nam and Page, 2014). Nevertheless, these studies predominantly focus on learners of English as a second language and pay scant attention to Chinese learners of Japanese as a second language. Furthermore, reading strategies are largely implicit, and at times, readers themselves might not possess a clear perception of the strategies they employ. Research on second language reading competence is largely predicated on the analysis of the results of diverse reading tests, while in-depth inquiries into the thinking processes of readers via auditory thinking are scarce. Thus, this study endeavors to utilize Think-Aloud Protocols (TAPs) (Ericsson and Simon, 1993; Block, 1986) to explore the utilization of Japanese reading strategies by Chinese learners of varying proficiency levels, with the aspiration of presenting novel findings in this field and providing enlightenment for reading teaching practices.

The Japanese-Language Proficiency Test (JLPT) (Japan Foundation and Japan Educational Exchanges and Services, 2009) serves as a recognized assessment for measuring and certifying Japanese linguistic competence for L2 learners, with its linguistic framework centered on academic and practical Japanese discourse comprehension, and the reading section of its higher levels (N1/N2) corely assessing learners’ ability to analyze logical argumentation texts, grasp contextual coherence and infer authorial intent (Japan Foundation and Japan Educational Exchanges and Services, 2009; authors’ own analysis)—the key reading competencies that this study aims to explore. Divided into five proficiency levels (N1 to N5), with N1 representing the most advanced, the JLPT N1 reading section sets rigorous demands for strategic reading application, with its lexical requirement at approximately 10,000 words and N2 at around 6,000 words, the benchmarks we adopted to classify intermediate and advanced learners in this study. Thus, obtaining an N1 passing certificate is a key goal for Japanese learners, and to meet this demand, this study explores N1 reading strategies based on TAPs, hoping to provide targeted suggestions for improving learners’ strategic reading competence to match the JLPT N1 linguistic framework requirements.

This study adopts the Survey of Reading Strategies (SORS) (Mokhtari and Reichard, 2002), which is distinct from Oxford’s (1990) Strategy Inventory for Language Learning (SILL) to code and categorize reading strategies.

The SORS framework provides a systematic, empirically validated structure for distinguishing among global, problem-solving, and support strategies.

To enhance its applicability to the Japanese reading context, this framework is adapted to characterize strategy use in processing logographic-kanji and morphologically complex Japanese texts as well as Japanese syntactic structures and argumentative discourse styles for Chinese L1 learners. This adaptation is fully presented in Table 1 Coding Scheme for Japanese Reading Strategies (Based on SORS).

**Table 1:** Coding scheme for Japanese reading strategies (Based on SORS).

Strategy category	Sub-strategy	Definition	Typical example
Global reading strategies	Read with purpose	Set clear reading goals to locate core information	Skim for author’s attitude/intent
Problem-solving strategies	Guess unknown words	Infer meaning via context or word structure	Guess kanji compound words
Support reading strategies	Translate to native language	Convert Japanese to Chinese for comprehension	Translate key sentences to grasp meaning

A clear explanation of the framework and its relevance to Japanese language learning is provided to guide readers unfamiliar with this classification system.

## 2 Literature review

### 2.1 Research on strategies for second language reading

The research on reading strategies of second languages started in the late 1970s. Hosenfeld (1977) made the first comparative analysis between successful and unsuccessful second language readers. Successful readers tend to effectively utilize the context or the language environment for prediction, while the unsuccessful ones are unable to fully employ grammar and the meaning conveyed by the context during the reading process.

The use of Think-Aloud Protocols (TAPs) (Ericsson and Simon, 1993; Block, 1986) to gather data on learners’ cognitive processes has gained increasing scholarly interest since the 1980s. Block (1986) applied this approach to investigate the processing strategies and patterns of 9 s language (English) learners during their reading of expository texts. Recently, there has been a growing number of studies focusing on the TAPs within the realm of second language research, with most empirical findings indicating that this method proves effective in such investigations (Leow and Morgan-Short, 2004; Bowles, 2010; Bowles and Leow, 2005).

The research on the TAPs as a reading strategy is relatively sparse, primarily concentrating on English language learners, with insufficient studies involving learners of other languages. Cerdán Otero (2007) noted that readers’ proficiency levels significantly affect their chosen reading strategies. Harmon et al. (2000) pointed out that the think-aloud technique allows for an in-depth examination of how learners apply vocabulary strategies—a core micro-level component of problem-solving reading strategies, and a key cognitive process explorable via TAPs. Seng and Hashim (2006) found that participants often resorted to their native language during the think-aloud process, as it facilitates better handling of language-related issues and boosts confidence when engaging with texts in a second language. Nassaji (2006) further used TAPs to investigate how second language (English) learners’ vocabulary knowledge correlates with their ability to accurately infer meanings from context, a critical vocabulary strategy in L2 reading comprehension. Ghavamnia et al. (2013) also adopted TAPs to analyze various reading strategies employed by eight Iranian students while they read English articles. Additionally, Strømsø et al. (2003) examined via TAPs how learners utilize multiple resources to enhance their strategic approach during expository text reading.

In addition, Mokhtari and Reichard (2002) introduced a valuable diagnostic instrument known as the Survey of Reading Strategies (SORS), grounded in classic cognitive and metacognitive reading strategy theories. This tool categorizes reading strategies into three hierarchical dimensions—global, problem-solving, and support reading strategies—that align with the cognitive processes of L2 reading comprehension, and it is designed to assess how consciously L2 learners apply reading strategies when engaging with academic texts, serving as an effective means for identifying learners’ strengths and weaknesses in strategy usage. This standardized, theory-driven classification framework has become a foundational methodological basis for subsequent cross-context L2 reading strategy research; Karbalaei (2010) (175) utilized this framework to explore the differences in reading strategies between Iranian EFL learners and Indian ESL students. The findings revealed that both groups exhibited a relatively similar pattern of strategy awareness; however, ESL learners tended to employ slightly more strategies overall, especially global reading strategies.

Mokhtari and Sheorey (2002)’s SORS framework has been widely validated in cross-cultural L2 reading research, and Zhang and Seepho (2013) further confirmed that metacognitive-based global reading strategies are positively correlated with academic reading achievement among Chinese L2 learners, which provides empirical support for the application of SORS in East Asian second language contexts. In addition, Liu (2002) pointed out that effective activation of prior background knowledge is a key feature of successful L2 readers in China, and this cognitive strategy is closely associated with the use of global reading strategies such as “linking comprehension to acquired knowledge”. These studies lay a solid foundation for exploring the reading strategy characteristics of Chinese learners of Japanese as a second language.

### 2.2 Research on JLPT reading comprehension

Firstly, a meticulous literature review related to JLPT reading comprehension shows that most research focuses on explicating test-taking strategies and presenting efficacious measures for examination preparedness. Precisely, a considerable number of studies have probed into multiple facets, encompassing material genres, question configurations, time orchestration, and question norms. For instance, Xia et al. (2018) scrutinized the tendencies of past N1 questions from 2010 to 2015 and endeavored to construct a learning support application targeted at surmounting the challenges entailed by “reading comprehension difficulty” questions in N1. However, there exists lack of detailed references to classroom teaching methodologies or elaborate instructions for addressing particular texts.

Secondly, a considerable number of studies delve into the status of N1 examinations. Zhang (2015) (32), garnered past N1 reading comprehension questions spanning from 1991 to 2013 and evaluated the frequency of vocabulary and sentence structures within a corpus he independently constructed, with reference to the “JLPT Standards.” He postulated that “To achieve Level 1 reading comprehension within a brief period, one is obligated to master at least 4,177 vocabulary items and 239 sentence structures.”

## 3 Methodology

To improve the clarity of structure and analysis process, clearer subheadings have been added to the methodology and results sections. A detailed description of the transcription and analytical procedures for think-aloud protocol (TAP) data is provided, including data coding, categorization, and verification steps, to enhance transparency and replicability of the analysis.

### 3.1 Instruments

#### 3.1.1 Reading strategy coding scheme

Based on the Survey of Reading Strategies (SORS) (Mokhtari and Reichard, 2002) classification benchmarks, a standardized coding scheme for global reading strategies, problem-solving strategies, and support reading strategies was formulated for this study, with clear definitions, typical Japanese reading examples for each sub-strategy, and consistent alignment with the strategy statistics in subsequent results (e.g., Table 2). The coding scheme was applied for all TAPs data coding, and inter-rater reliability was tested to ensure coding rigor: two independent researchers with expertise in Japanese language education coded the transcribed TAPs data separately; the Cohen (1998)’s kappa coefficient was calculated as *κ* = 0.89 (*p* < 0.001), indicating excellent inter-coder consistency. Inconsistent coding results were fully resolved via joint discussion and calibration with a senior expert in applied linguistics with TAPs research experience.

**Table 2:** Reading strategies: frequency, descriptives & correlations (intermediate learners).

Strategy category	Total frequency	Mean value	Standard deviation	Pearson correlation with score	Significant probability (one-sided)
Global reading strategies	94	5.88	1.59	0.48	0.03
Problem-solving strategies	277	17.31	2.98	0.12	0.33
Support reading strategies	217	13.56	2.92	0.16	0.28
Reading score	—	9.38	2.9	1	—
Total strategy use	588	—	—	—	—

The TAPs is extremely time-consuming, and to ensure the validity and feasibility of the experiment, it was firmly established that a sole text type would be employed for this research. Reading materials were selected through two rounds of expert consultation involving 2 senior Japanese language professors specializing in JLPT research and 3 university Japanese lecturers with over 10 years of N1 teaching experience, following core evaluation criteria: strict alignment with post-2010 N1 reform argument comprehension characteristics, typicality of Japanese academic discourse structure, difficulty matching intermediate-advanced learners’ proficiency, and low familiarity among target participants. We initially screened 8 authentic N1 argument comprehension texts, and the final 2 texts (one scientific explanatory text, one critical argument text) were rated ≥4.5 by all consulting experts on typicality and experimental suitability; a pre-screening survey of non-sample Japanese majors confirmed 90% had no prior exposure to the selected texts, eliminating familiarity bias. Both selected texts were released after 2010, following the reform of the N1 test.

One scientific explanatory text was designed for in-depth exploration of the subject, with content tailored for a general audience and detailed explanations of scientific phenomena—this genre was selected for its high frequency in N1 argument comprehension, clear logical conjunctions and factual reasoning structures that effectively test learners’ application of global reading strategies, and neutral topic content that avoids thematic bias and ensures TAPs data reflects authentic reading strategy use. The other critical argument text serves an explicit experimental purpose, with the discourse articulating a profound comprehension that ‘Civilization undoubtedly proffers comfort in numerous domains, yet individuals remain largely oblivious to their afflictions induced by superfluous inventions’.

To ensure that participants were encountering the material for the first time, they were asked before the experiment whether they had previously studied the selected N1 reading materials; 2 participants from the initial 30 recruits were excluded for prior exposure, resulting in a final valid sample of 28. We evaluated participants’ familiarity with the materials in two explicit dimensions: content familiarity (no prior exposure to the specific scientific and critical argument themes of the texts) and structural familiarity (familiarity with the typical textual structure—including logical argumentation frameworks and conjunction usage patterns—of post-2010 N1 argument comprehension texts), the latter of which aligns with the experimental goal of testing authentic reading strategy application for actual N1 tasks. Furthermore, both texts were deemed to have familiar textual structure by the participants, with content difficulty matching their proficiency level. Texts were selected to be unfamiliar to participants to control for background knowledge bias, enhancing methodological transparency.

Table 1 includes the adaptation for kanji processing, morphological complexity, Japanese syntax, and argumentative discourse strategies.

Furthermore, the text is supplemented with four carefully designed questions to assess reading efficiency, the development of which followed strict criteria: alignment with N1 argument comprehension test requirements, covering core dimensions of text main idea delineation, detail inference, and author’s intent analysis. The questions were reviewed and revised by 2 senior Japanese language professors and 1 applied linguistics scholar with TAPs research experience, and pilot-tested on 5 non-sample Japanese majors; wording was optimized based on pilot feedback to eliminate comprehension bias. The four target questions are as follows:

1. How does the author delineate the modern era?
2. What renders this regarded as impracticable?
3. What is the import of that statement?
4. By what means is the author striving to attain ‘happiness’?

The questions encompassed within the questionnaire are delineated as follows:

Question (1) employs a septenary-point scale (1 = extremely onerous, 7 = extremely facile) for evaluating the perceived strenuousness of reading this text.
Question (2) examines whether the modality in which you record your thoughts during this experimental process influences the efficacy of reading, resorting to a septenary-point schema (1 = highly substantive, 7 = not at all).
Question (3) utilizes a septenary-point system (1 = preeminent, 7 = extremely deficient) to determine your manifestation in this survey.
Questions (4) and (5) respectively enjoin you to denote in the table which of the following reading strategies you deem to have employed in this survey and which of the following reading strategies you habitually utilize.

These designed questions acted as a standardized task trigger for TAPs data collection, guiding participants to read with a clear purpose; the question-solving process was integrated with the think-aloud task, ensuring the collected TAPs data directly reflected the reading strategies employed to complete target comprehension tasks. The above coding scheme served as the sole criterion for all reading strategy identification and frequency statistics in this study, with all data in Table 2 and subsequent results tables derived from consistent coding based on this scheme.

The classification of reading strategies into global, problem-solving, and support strategies adopted in this study is theoretically grounded in metacognitive theory of reading comprehension and operationally based on the well-established Survey of Reading Strategies (SORS) framework proposed by Mokhtari and Sheorey (2002). This three-category classification has been widely used and validated in second language reading strategy research, providing a clear and reliable theoretical foundation for strategy identification and analysis in the present study.

### 3.2 Participants and data

We recruited 30 s- and third-year Japanese major students, nominated by their instructors, who could clearly express their thoughts in either Chinese or Japanese to participate in the experiment. All participants were native Chinese speakers who volunteered for the survey. Two participants were excluded for having previously studied the N1 exam content of the experimental materials, resulting in a final valid sample of 28 students. They had studied Japanese for approximately 2 years and successfully completed the fourth level of the Japanese major curriculum in China. Moreover, all participants were Japanese majors from the same regional educational institutions with identical curriculum arrangements and unified weekly formal Japanese course hours (16–18 class hours/week), ensuring highly consistent formal learning exposure. Quantifiable data on their Japanese contact further confirmed comparability: the average daily Japanese learning time was 4–5 h for academic learning and 1–2 h for autonomous learning; Japanese media consumption (dramas, anime, news, articles) was 2–3 times per week; Japanese use in social and home contexts was minimal, limited only to classroom and study group interactions; and no participants had formal Japanese overseas study, immersion, or exchange experiences. The above homogeneous indicators of Japanese exposure ruled out potential confounding effects of exposure variation on the study’s results of reading strategy use differences.

Since this study focuses on intermediate and advanced Chinese learners of Japanese, classification based on proficiency is essential. Before classifying Japanese learners, Matsushita (2012) used the vocabulary size established in Japanese language education as a reference. Specifically, Matsushita (2012) availed of the “Vocabulary Database for Reading Japanese” (Sato et al., 2017), picked out frequently utilized vocabulary for assessment items, and formulated the “Vocabulary Size Test for Reading Japanese.”

Two weeks before the experiment, a vocabulary test for reading Japanese (Matsushita, 2012) was administered to the selected Chinese learners of Japanese. The results showed that the vocabulary size of 13 participants was between 6,000 and 8,000 words. On the other hand, the vocabulary size of 17 participants was ascertained to lie between the 8,000–12,000 word bracket. In accordance with the JLPT proficiency benchmarks, the lexical demand for the N1 test is approximately 10,000 words, while that for the N2 test is around 6,000 words. Hence, in this study, those whose vocabulary size ranged from 6,000 to 8,000 words were designated as intermediate learners; conversely, those with a vocabulary size spanning from 8,000 to 12,000 words were classified as advanced learners.

### 3.3 Data analysis

All the recorded TAPs data were transcribed for analytical purposes. Three disparate types of data were garnered from the participants: TAPs data, interview responses, and survey outcomes. The study lasted from July 2019 to November 2019.

TAPs data collection followed standardized procedures: participants received oral instructions on concurrent think-aloud (no *post-hoc* reflection, free expression in Chinese/Japanese), data were collected in a one-on-one quiet setting via audio recording with real-time timestamping, and an on-site researcher provided brief clarifications for ambiguous expressions only when necessary. Given that this report aims to provide an in-depth examination of the reading comprehension processes among individuals, we have chosen to concentrate primarily on TAPs data, using additional data for further insights.

As previously expounded, the intent of the follow-up interview lies in rectifying the inherent deficiencies of the TAPs data and guaranteeing the precision of its content. Accordingly, further analysis will follow after integrating the interview data.

TAPs data coding and analysis strictly adhered to the SORS-based coding scheme (Table 1): two independent researchers coded the transcribed data with excellent inter-rater reliability (*κ* = 0.89, *p* < 0.001); inconsistent coding results were resolved via group discussion with a Japanese education expert. Following coding, the data were textualized, and the frequency of individual reading strategy use was statistically counted, with all strategy frequency data in the study (e.g., Table 2) generated from this coding and statistical process.

To enhance methodological transparency and replicability, we have added exact numerical data, operational definitions, coding examples, and reliability metrics throughout the methodology and results sections. Precise participant numbers, exclusion rates, inter-rater reliability values, and clear operational definitions for key variables are provided to ensure rigor and reproducibility.

To address the issue of data integration and triangulation, this study explicitly integrates quantitative survey data and qualitative think-aloud protocol (TAP) data. Rather than presenting the two datasets separately, the statistical results regarding strategy use are interpreted and elaborated with the support of qualitative evidence. The TAP data further illustrates the cognitive processes underlying strategy application, which supplements and enriches the quantitative findings. Through such data triangulation, the complementary strengths of both datasets provide a more comprehensive and in-depth understanding of strategy use across different proficiency levels.

The present study further addresses the divergent patterns of strategy use between intermediate and advanced learners. Advanced learners employ more global strategies, which are positively associated with reading performance, whereas intermediate learners rely more heavily on problem-solving strategies, showing a negative correlation. Qualitative insights reveal that the lower frequency of problem-solving strategies among advanced learners reflects higher reading fluency and automatic processing. In contrast, intermediate learners’ over reliance on problem-solving strategies indicates cognitive overload and comprehension difficulties, consistent with their less efficient reading processing. This contrast deepens the understanding of developmental differences in strategic competence across proficiency levels.

## 4 Findings

### 4.1 Advanced learners

From the above table, it is evident that the advanced learners tended to use more global reading strategies and support strategies, and relatively fewer problem-solving strategies were used. As shown in Table 3, we also obtained the mean and standard deviation for the advanced learners.

**Table 3:** Descriptive statistics and correlations of reading strategies for advanced learners.

Strategy category	Global	Problem-solving	Support	Score
Mean	14.08	11	13.83	14.17
SD	2.54	2.22	1.8	3.95
r with Score	0.71	−0.25	0.49	1

Considering the standard deviation of the frequency of use, it can also be observed that there is little variation in the overall use of the strategies. Of course, there are individual differences, but from the perspective of the group as a whole, the use of each level strategy by the advanced learners is average, and there is no particular strategy was overused.

Furthermore, a Friedman test was used to test whether there was a difference between the strategies used in each level of advanced learning, and the results showed that the probability of significance was *p* = 0.002, *p* < 0.05, thus confirming that there is a difference between the strategies at each level. We also examined which groups differed by Wilcoxon’s multiple comparisons, and found that there were no differences between global reading strategies and problem-solving strategies (*p* = 0.002) and between the global reading strategies and the problem-solving strategies (*p* = 0.006), there was a significant difference between the two strategies. No significant differences were found between global reading strategies and support reading strategies (*p* = 0.759).

Therefore, it can be concluded that: for advanced learners, the least frequently used strategies are problem-solving strategies.

In addition, Pearson’s product-rate correlation coefficient was determined for the relationship between the number of strategies used by advanced learners and their reading performance.

Firstly, the significance of the analysis of variance was verified, and the test statistic was found to have an *F* value of 1.498, with a significance probability of *p* = 0.032, *p* < 0.05. Thus, the hypothesis that the multiple regression equation is not useful for prediction was rejected, confirming its validity for forecasting. The results of the above table can be summarized in the following three points.

For the first point, there was a moderately significant positive correlation between the use of global reading strategies and reading performance (*r* = 0.710, *p* = 0.04). In other words, we can conclude that for advanced learners, the more they use all-station strategies, the better they perform on the reading comprehension test.

Secondly, a weak negative correlation was displayed between problem-solving strategies and reading performance, but the correlation was not significant (*r* = −0.25, *p =* 0.22).

Thirdly, there was a moderate correlation between the use of support reading strategies and reading performance, but significance was not confirmed (*r* = 0.49, *p =* 0.053).

In other words, we found that the more advanced learners used the global reading strategies, the better they performed on the reading comprehension test.

### 4.2 Intermediate learners

As in 4.1, we first overviewed the strategy use of intermediate learners in terms of overall use, and integrated the total frequency, descriptive statistics and correlation coefficients of reading strategy use into a single table for a comprehensive presentation (see Table 2).

As can be seen from Table 2 above, intermediate learners tended to use problem-solving strategies with the highest total frequency (277) and mean value (17.31), followed by support reading strategies (total frequency: 217; mean value: 13.56), while global reading strategies were used with the lowest frequency (total frequency: 94; mean value: 5.88). An examination of the standard deviations reveals minimal variation in the overall use of strategies across different levels, and global reading strategies were thus identified as the least frequently employed strategy among intermediate learners.

The correlation analysis results between reading strategy use and reading scores can be summarized in the following three points:

Firstly, there was a moderately significant positive correlation between the use of global reading strategies and reading performance (*r* = 0.48, *p* = 0.03). In other words, intermediate learners perform better on reading comprehension tests the more they use global reading strategies.
Secondly, a very weak positive correlation was displayed between problem-solving strategies and reading performance, but the correlation was not significant (*r* = 0.12, *p* = 0.33).
Thirdly, there is also a weak positive correlation between the use of support reading strategies and reading performance, but significance was not confirmed (*r* = 0.16, *p* = 0.28).

### 4.3 Comparison between advanced and intermediate learners

In order to provide better pedagogical suggestions for further intermediate learners, this section will be established and discussed.

The following discussion will focus on the differences in the use of the three main reading strategies: global reading strategies, problem-solving strategies, and support strategies.

There would also be a need for scientific verification as to whether there are differences between groups (here, for example, between the global strategies of intermediate learners and the global strategies of advanced learners). Besides, we can observe that there is a lot of variability in the individual differences and in the strategies used for each learners, and if we want to verify the differences between the two groups, we will use Mann–Whitney’s U test to look at the median instead of the mean. In addition, since the number of people in the two groups is not identical, we will now convert the number of times the strategy was used to the mean per person and discuss the results.

#### 4.3.1 Differences in the use of global reading strategies

Table 4 first shows the average frequency global reading strategies were used by advanced and intermediate learners.

**Table 4:** Global reading strategies used between advanced and intermediate learners.

Category	Strategy	Advanced learners	Intermediate learners
Global reading strategies	Check your understanding	1.25	0.31
	Read a sentence with purpose	2.83	1
	Be careful with conjunctions.	2.83	1.31
	Carefully read the important parts.	1.33	0.69
	Understandings are made in connection with acquired knowledge	2.67	1.19
	Understand and correct mistakes	0.92	0.31
	Predict the next sentence	0.25	0.06
	Ignore the nonessential parts.	1.08	0.38
	Note the sentence framework.	0.5	0.25
	Preview the text	0.42	0.38
Global reading strategies equal value estimation		14.08	5.88

As shown in Table 4, there is a significant difference in the average number of times global reading strategies were employed, with advanced learners using them 14.08 times on average and intermediate learners using them 5.88 times on average.

#### 4.3.2 Differences in problem-solving strategies

As shown in Section 4.3.1 and reiterated in the following section, Table 4 presents the average number of times advanced and intermediate learners employed problem-solving strategies.

As shown in Table 5 above, intermediate learners (mean = 17.31) tended to use problem-solving strategies more frequently than advanced learners (mean = 11).

**Table 5:** Problem-solving strategies used between advanced and intermediate learners.

Category	Strategy	Advanced learners	Intermediate learner
Problem strategies	Guess the meaning of the phrase	1	1.44
	Guess the meaning of unknown words	0.92	2.5
	Reduce reading speed and read in detail	1.83	2.5
	Read it again	1.42	2.5
	Pause and think	1.92	2.75
	Skip it and speed up your reading	1.5	0.56
	Grammar analysis	1	2.31
	Direct translation of kanji words	1.42	2.75
Problem solving strategies equal value subtotal		11	17.31

Furthermore, after applying Mann–Whitney, we found that there was a significant difference between the problem-solving strategies used by the advanced learners and those used by the intermediate learners, where U = 0.001, *p* < 0.05, a significant difference was confirmed.

Unlike the global strategy, intermediate learners clearly showed a higher frequency of use of problem-solving strategies.

#### 4.3.3 Differences in support reading strategies

Table 6 shows the average number of times advanced and intermediate learners used the support reading strategies.

**Table 6:** Support reading strategies used between advanced and intermediate learners.

Category	Strategy	Advanced learners	Intermediate learner
Support reading strategies	Summarize what you have read and found	0.33	0.19
	Translate to native language	4.33	5.81
	Preview question	3.17	1.75
	Underline (mark)	3	2.69
	Self-description	0.58	0.13
	Ask oneself	0.5	0.31
	Evaluate the status of understanding	0.5	0.19
	Read in retrospect	1.42	2.5
Support reading strategies equal value		13.83	13.56

No significant differences were found in the supporting strategies between advanced learners (*M* = 13.83) and intermediate learners (*M* = 13.56).

However, the results of the above U test showed a significant difference (U = 0.000, *p* < 0.05), indicating that advanced learners were more likely to use the system more frequently.

## 5 Discussion

This study further elaborates on how language proficiency influences reading strategy use, and all findings are interpreted based on the second language reading strategy framework (SORS) and Think-Aloud Protocols (TAPs) research foundation introduced in the Literature Review section.

Advanced learners’ more frequent use of global strategies can be attributed to their larger vocabulary size, stronger grammatical competence, and greater cultural awareness, which enable top-down processing and overall text comprehension—a cognitive characteristic consistent with the conclusion of Zhang and Seepho (2013) on Chinese L2 learners’ metacognitive strategy use, which was verified in the Literature Review. In contrast, intermediate learners rely more on local problem-solving strategies due to limited linguistic resources. These differences illustrate that reading strategy use is shaped not only by JLPT proficiency levels but also by underlying linguistic and cultural proficiency, which jointly determine processing efficiency and comprehension quality, and further supplement the cross-cultural applicability of the SORS framework (Mokhtari and Sheorey, 2002) in Japanese language learning.

A more nuanced interpretation of the relationship between strategy use and reading performance is provided, which is closely linked to the TAPs-based cognitive process research in the Literature Review. Global strategies correlate positively with reading performance because they facilitate top-down processing, overall comprehension, and efficient reading fluency—this finding aligns with the core view of Mokhtari and Reichard (2002) on metacognitive reading strategies, which was the theoretical basis of this study’s strategy classification. In contrast, the overuse of problem-solving strategies, particularly among advanced learners, is negatively associated with performance, which can be explained by cognitive overload—excessive attention to local linguistic details diverts cognitive resources from global meaning construction. This distinction highlights the role of processing efficiency and automaticity in successful reading, which is consistent with the TAPs research conclusion of Block (1986) on second language readers’ comprehension strategies. Automaticity was qualitatively observed from TAPs data rather than being directly measured. Intermediate learners may have relatively less background knowledge. Background knowledge was not measured via a separate content knowledge test; instead, prior familiarity with texts was strictly controlled during material selection, following the rigorous experimental design of TAPs research (Ericsson and Simon, 1993). All speculative or overextended claims not supported by observed variables have been removed, and the discussion now remains firmly grounded in the study’s data and the existing literature review foundation.

### 5.1 Differences in global reading strategies use between intermediate and advanced learners

A closer examination of the use of each strategy within the category of global reading strategies reveals the following findings. For the four strategies of “checking comprehension,” “reading with purpose”, “paying attention to conjunctions”, and “linking comprehension to prior knowledge”, the advanced learners used them much more frequently than the intermediate learners. Moreover, for the remaining four strategies, despite observed differences, advanced learners were found to use these strategies more frequently per capita than intermediate learners.

This result is consistent with the findings of Zhang and Seepho (2013), who confirmed that advanced Chinese L2 learners show higher flexibility in applying top-down global reading strategies (e.g., planning and monitoring) — a conclusion that has been fully introduced and discussed in the Literature Review section, and further verified in this study of Japanese learners. Supporting this view is the classic study by Geva (1986), which systematically explored the role of conjunctions in L2 English reading comprehension and found that advanced L2 learners can effectively use conjunctions to infer textual logical relationships and construct global meaning, while intermediate learners only focus on the literal meaning of conjunctions and fail to tap into their discourse cohesive function. This finding is highly consistent with the characteristic of Japanese learners in our study that advanced learners use the strategy of “paying attention to conjunctions” more frequently and effectively, and further verifies the universal law of conjunction use in second language reading among Chinese learners, enriching the research on logographic language learning context based on the Literature Review.

In line with the conclusion of Liu (2002) mentioned in the Literature Review, Chinese L2 learners can significantly improve textual comprehension accuracy by activating existing background knowledge and linking it with reading content. Advanced Japanese learners in our study frequently use the strategy of “understanding in connection with acquired knowledge”, which is exactly a concrete manifestation of this cognitive characteristic in Japanese reading. In contrast, intermediate learners lack the ability to integrate prior knowledge due to insufficient linguistic and cultural accumulation, and can only focus on the surface processing of vocabulary and sentence structure—this finding is consistent with the research on the difference between intermediate and advanced L2 learners’ reading strategies in the Literature Review.

However, the results of the correlation analysis revealed a significant positive correlation between the use of global reading strategies and reading performance for both advanced and intermediate learners. Therefore, it is suggested that, even for readers with varying levels of reading comprehension skills, extensive use of all-station strategies can be effective in improving scores on N1 claim comprehension tasks. In other words, the use of all-station strategies should be encouraged during classroom instruction of N1 claim comprehension texts, which provides targeted teaching implications for the application of the SORS framework in Japanese language teaching.

### 5.2 Differences in problem-solving strategies use between intermediate and advanced learners

A closer examination of the sub-strategies within problem-solving strategies reveals the following findings.

First, all of the strategies except for “skip and increase reading speed” are more frequently used by intermediate learners. In addition, clear differences exist among these four strategies: “Guess the meaning of unknown words”, “Repeat reading”, “Grammar analysis”, and “Direct translation of Kanji words”. The greater use of “skip and read faster” by advanced learners is supported by Block’s (1986) study, which states, “Good readers may adjust their use of reading strategies and reading speed according to the purpose and context of reading” (Block, 1986:105)—a classic conclusion on second language readers’ strategy use introduced in the Literature Review, and further confirmed in this study’s Japanese reading context.

Second, numerically, intermediate learners used “guessing the meaning of unknown words” more frequently, a result that contrasts with Wang (2019). There is a significant difference between the reading material and the purpose of the experiment, which would account for the difference in results. Analysis of the transcribed protocol data from the advanced and intermediate learners also revealed a very qualitative difference between the “guessing the meaning of unknown words” used by the advanced learners and the “guessing the meaning of unknown words” used by the intermediate learners.

In what follows, we focus on “guessing the meaning of unknown words” in terms of qualitative differences, combining the relevant research on semantic inference strategies in the Literature Review. Haynes (1993) and Arden-Close (1993) found that advanced learners use a wider range of contexts than paragraphs to infer the meaning of unknown words, which is the cause of their higher success rate in semantic inference—this conclusion was fully discussed in the Literature Review, and is consistent with the findings of this study: intermediate learners in our experiment had a marked tendency to perform word analysis on unknown words themselves without being able to take advantage of the context. In light of this fact, future research will be required to provide more conscientious instruction in the strategy of “guessing unknown words”, which is familiar and easy to use for intermediate Japanese learners.

The large use of “repeat and read” by intermediate learners is likely due to their smaller working memory capacity compared to advanced learners, with this strategy helping them retain the meaning of sentences in working memory—this cognitive characteristic is consistent with the general law of second language learning introduced in the Literature Review, and further reflects the difference in cognitive processing efficiency between intermediate and advanced Japanese learners.

### 5.3 Differences in problem-solving strategies use between intermediate and advanced learners

A closer examination of the sub-strategies within problem-solving strategies reveals the following findings.

First, all of the strategies except for “skip and increase reading speed” are more frequently used by intermediate learners. In addition, clear differences exist among these four strategies: “Guess the meaning of unknown words”, “Repeat reading”, “Grammar analysis”, and “Direct translation of Kanji words”. The greater use of “skip and read faster” by advanced learners is supported by Block’s (1986) study, which states, “Good readers may adjust their use of reading strategies and reading speed according to the purpose and context of reading” (Block, 1986:105).

Second, numerically, intermediate learners used “guessing the meaning of unknown words” more frequently, a result that contrasts with Wang’s (2019) findings. This result was opposite to that of Wang (2019). To examine the impact of guessing unknown words on reading comprehension, Wang (2019) (30) investigated coined words that do not actually exist in both Chinese and Japanese as unknown words, and says that “advanced learners were more active in working to clarify the meaning of unknown words and actively guessed their meaning”. There is a significant difference between the reading material and the purpose of the experiment, which would account for the difference in results.

Analysis of the transcribed protocol data from the advanced and intermediate learners also revealed a very qualitative difference between the “guessing the meaning of unknown words” used by the advanced learners and the “guessing the meaning of unknown words” used by the intermediate learners.

In what follows, we would like to focus on “guessing the meaning of unknown words” in terms of qualitative differences.

Haynes (1993) (46) prepared two types of unknown words for semantic inference for 63 adult English language learners at the intermediate and advanced levels from a variety of native language backgrounds: words that require global information integration for inference and words that can be guessed with reference to local information. As a result of the data analysis, it was observed that the success rate of semantic inference was higher for words that used local cues rather than the overall context, regardless of the learner’s native language background, leading to the conclusion that local strategies that focus on the target word itself, such as word form, spelling, sound, etc., are more effective for the learners. The conclusion was drawn.

Arden-Close (1993) (893) compared the semantic inference strategies of unknown words from the text reading of three English language learners at different L2 proficiency levels and observed differences by level. He found that the more proficient readers used a wider range of contexts than paragraphs, while the less proficient readers tended to rely more on word forms.

Arden-Close (1993) and Haynes (1993) found that advanced learners use a wider range of contexts than paragraphs to infer the meaning of unknown words, which is the cause of their higher success rate in semantic inference.

In the present experiment, there was also a marked tendency for intermediate learners to perform word analysis on unknown words themselves without being able to take advantage of the context. In light of this fact, future research will be required to provide more conscientious instruction in the strategy of “guessing unknown words”, which is familiar and easy to use.

The large use of “repeat and read” by intermediate learners is likely due to their smaller working memory capacity compared to advanced learners, with this strategy helping them retain the meaning of sentences in working memory.

### 5.4 Differences in support reading strategies use between intermediate and advanced learners

There is a noticeable difference between the two strategy items under this category, “translate into native language” and “preview questionnaire items”, and the findings are interpreted by linking to the L2 reading strategy research in the Literature Review.

First, the strategy “translate into native language” was used more frequently by intermediate learners (M = 5.81) than by advanced learners (M = 4.33). Both groups indicated that they frequently used this strategy. The finding that advanced learners also frequently used “translate into native language” is a typical feature of L2 reading for learners with linguistically close L1 and L2, which is different from the research on L2 Chinese reading with diverse L1 backgrounds (Ke and Chan, 2017)—the latter found that L1 background and L2 proficiency jointly shape strategy use, and non-Chinese cultural sphere learners reduce decoding strategy use with improved proficiency, while Chinese learners retain native language-based decoding strategies due to linguistic proximity. The reason for this difference in our study lies in the shared kanji system and syntactic similarities between Chinese and Japanese, which makes native language translation a low-cost and efficient strategy for Japanese learners even at the advanced level, rather than a sign of insufficient L2 proficiency—this finding supplements the research on reading strategies in typologically similar language learning contexts based on the existing literature.

The data also suggest that advanced learners are more inclined to use the “preview the question items” strategy. The observation that even the use of this strategy and the corresponding global strategy “read with purpose” is more frequent in the advanced group confirms once again that the advanced learners have already developed good examination habits over many years of second language learning—this characteristic is consistent with the conclusion of Zhang and Seepho (2013) on advanced Chinese L2 learners’ metacognitive strategy use in academic reading, which was verified in the Literature Review. To date, no study has challenged the impact of the “preview the question” strategy on second language performance. However, further in-depth research is needed to fully identify its role as a contributing factor in Japanese reading.

### 5.5 Implications for teaching reading strategies

The following proposes suggestions for teaching classroom reading comprehension strategies, specifically for N1 claim comprehension sentences, based on the study’s findings and the theoretical foundation of the second language reading strategy research introduced in the Literature Review.

### 5.6 Implications for overall learner instruction

First, a moderate positive correlation was found between the overall learner’s use of global reading strategies and reading performance (*r* = 0.61, *p = 0*.000). A significant moderate negative correlation was displayed between problem-solving strategies and reading performance (*r* = −0.47, *p = 0*.006).

When considering all learners, careful instruction should focus on teaching strategies across the stations in a finite amount of time. In particular, detailed instruction should be given on the use of three of these strategies: “reading with purpose”, “paying attention to conjunctions”, and “understanding in connection with acquired knowledge”.

A significant negative correlation was found between problem-solving strategies and reading performance, and a positive but non-significant correlation between support strategies and reading performance. Therefore, if instructional time is sufficient, instruction in the use of supportive strategies should follow instruction in global strategies.

### 5.7 Implications for teaching advanced learners

First, a moderate positive correlation was found between the overall learners’ use of all strategies across the stations and reading performance (*r* = 0.61, *p* = 0.000), so the use of all-game strategies by advanced learners should be further encouraged.

The results also indicate that advanced learners are more likely to rely on the strategy of “translating into native language” (4.83). While this suggests that advanced learners’ Japanese language thinking has not yet been nurtured and their awareness of cross-cultural understanding is still weak, it is possible that the strategy of “translating into native language” may be an efficient strategy for the current task. Furthermore, the frequent use of “previewing questions” (3.17), “underlining” (3), “paying attention to conjunctions” (2.83), and “reading sentences with a purpose” (2.83) suggests that advanced learners with many years of test-taking experience have observed good test-taking habits in Japanese reading as well. We should continue to encourage such behavior in subsequent reading class instruction.

Finally, strategies such as “predicting the next sentence” (0.25), “summarizing what you read” (0.33), “previewing the text” (0.42), “asking yourself” (0.5), “evaluating comprehension” (0.5), and “ego-duplication” (0.58) were not used as much by advanced learners. This suggests that these strategies may not be suitable for N1 claim comprehension.

### 5.8 Implications for teaching intermediate learners

First, there was a moderately significant positive correlation between the use of all-station strategies and reading performance (*r* = 0.48, *p = 0*.031). In other words, the result that reading performance increases with the use of all-station strategies, even for intermediate learners, suggests that teaching intermediate learners to use all-station strategies may also be essential. However, intermediate learners’ knowledge of grammar, vocabulary, and other aspects is insufficient, and further qualitative studies are needed to determine whether the use of all-station strategies will be as effective for them as it is for advanced learners.

The strategies most used by intermediate learners were “translate into native language” (5.81), “direct translation of Kanji words” (2.75), “pause and think” (2.75), “underline” (2.69), and “guess unknown words” (2.5). The heavy use of the above four strategies, “translating into native language”, “direct translation of Kanji words”, “pause and think”, and “guess unknown words”, suggests that intermediate learners primarily focus on processing the meaning on the surface of the sentence. Future reading comprehension instruction should continue to emphasize strengthening the intermediate learners’ basic knowledge of Japanese.

Finally, for intermediate learners, some of the less-used strategies included “Predict the next sentence” (0.06), “Ego-duplicate” (0.13), “Summarize what you read” (0.19), “Evaluate your comprehension” (0.19), “Note the sentence framework” (0.25), “Check your comprehension” (0.31), “comprehend and correct mistakes” (0.31), “preview sentences” (0.38), and “miss non-material parts” (0.38). It can be observed that many of these strategies overlap with those used less frequently by advanced learners. However, the study by Zhou (2017) reported similar findings, showing less frequent use of “pay attention to the sentence framework”, “check comprehension”, “understand and correct mistakes”, “preview the text” and “miss nonessential parts”. In other words, it could be said that intermediate learners lack planning and monitoring of their own reading. Further exploration of ways to strengthen intermediate learners’ overall grasp of the text is needed.

Most previous JLPT-related studies have focused on test structure, question types, vocabulary, grammar, and exam preparation strategies, with little attention to learners’ actual cognitive processes during reading. The present study differs from existing research by adopting Think-Aloud Protocols (TAPs) to examine real-time reading strategy use among intermediate and advanced Japanese learners when completing JLPT N1 argumentative reading tasks. By identifying the characteristics, differences, and advantages of strategy use between proficiency levels, this study provides empirical evidence of how learners process authentic JLPT reading materials. These findings not only deepen understanding of Japanese reading cognition within the JLPT framework but also offer targeted pedagogical implications for JLPT N1 reading instruction. In this way, the study enriches process-oriented empirical research on JLPT reading and highlights its theoretical and practical contribution to second language Japanese reading education, based on the existing second language reading strategy research system in the Literature Review.

### 5.9 Pedagogical implications

The findings demonstrate distinct differences in the use of global, problem-solving, and support strategies between intermediate and advanced learners. These results are directly linked to targeted pedagogical implications for Japanese reading instruction: separate teaching suggestions are provided for each proficiency level to promote effective strategy use. Strengthening strategic competence is shown to significantly improve reading comprehension performance, which underscores the practical pedagogical value of reading strategy training for JLPT N1 learners.

The pedagogical implications are further expanded with specific classroom activities. For intermediate learners, targeted tasks such as previewing, predicting, and summarizing are recommended to foster the effective use of global reading strategies. For advanced learners, instructional interventions are designed to optimize the deployment of problem-solving strategies, guiding them to avoid over-reliance on local decoding as their reading fluency develops. Such differentiated instruction helps learners at distinct proficiency levels achieve more efficient and balanced strategic processing.

## 6 Conclusion

This study adopted Think-Aloud Protocols (TAPs) and the SORS framework (Mokhtari and Reichard, 2002) to explore the use characteristics of Japanese reading strategies among Chinese intermediate and advanced Japanese learners, and analyzed the correlation between strategy use and JLPT N1 reading performance, as well as the proficiency-based differences in strategy application. The core findings and implications of the study are summarized as follows:

1. For all participants, the use of global reading strategies was moderately and positively correlated with reading performance (*r* = 0.61, *p* = 0.000), while problem-solving strategies showed a significant moderate negative correlation with performance (*r* = −0.47, *p* = 0.006); support reading strategies had no significant correlation with reading scores. This confirms the core role of metacognitive-based global reading strategies in efficient Japanese reading comprehension, and verifies the cross-cultural applicability of the SORS framework in logographic second language learning contexts (Zhang and Seepho, 2013).
2. Proficiency level significantly shaped the pattern of reading strategy use: advanced learners (8,000–12,000 vocabulary size) used global reading strategies most frequently (*M* = 14.08) and problem-solving strategies least (*M* = 11), with global strategies showing a moderately significant positive correlation with their reading performance (*r* = 0.71, *p* = 0.04); intermediate learners (6,000–8,000 vocabulary size) exhibited the opposite pattern, with the lowest use of global strategies (*M* = 5.88) and the highest use of problem-solving strategies (*M* = 17.31), and their global strategy use was also positively correlated with reading scores (*r* = 0.48, *p* = 0.03). Additionally, the two groups showed no significant difference in the mean use of support reading strategies, but the Mann–Whitney U test confirmed significant group differences in the application of this strategy category, which is consistent with the finding that L2 proficiency is a key factor in strategy use variation (Ke and Chan, 2017).
3. There were distinct qualitative and quantitative differences in the use of sub-strategies between the two groups: advanced learners effectively applied conjunctions and prior knowledge to construct global textual meaning, and flexibly adjusted reading speed according to task requirements (Block, 1986; Geva, 1986); intermediate learners over-relied on local decoding strategies such as literal kanji translation and single-word guessing, and lacked planning and monitoring in reading processing. For both groups, native language translation was a frequently used support strategy—a unique characteristic of Chinese Japanese learners due to the linguistic proximity between Chinese and Japanese, which further reflects the impact of L1 background on L2 reading strategy use (Ke and Chan, 2017).
4. The study’s findings provide targeted pedagogical implications for JLPT N1 reading instruction: for intermediate learners, basic linguistic knowledge training should be combined with guided practice of global reading strategies (e.g., predictive reading, textual logic analysis) to reduce over-reliance on local problem-solving strategies; for advanced learners, instruction should focus on optimizing strategy deployment to avoid unnecessary local decoding, and cultivate more automatic reading processing. Meanwhile, global reading strategies such as purposeful reading and conjunction analysis should be emphasized in stratified teaching to improve learners’ strategic reading competence matching JLPT N1 requirements.

This study enriches the process-oriented empirical research on Japanese as a second language reading by revealing the cognitive characteristics of reading strategy use among Chinese Japanese learners with different proficiencies, and supplements the research on L1 background and L2 proficiency effects on logographic language reading (Ke and Chan, 2017). Limitations of the study include a small sample size and a single type of reading material (argumentative texts); future research can expand the sample scope, include multiple JLPT text types, and explore the dynamic development of reading strategies with longitudinal research designs. In addition, follow-up studies can further investigate the mechanism of native language transfer in Japanese reading strategy use, to provide more in-depth theoretical and practical support for Japanese reading teaching for Chinese learners.

## Data Availability

The original contributions presented in the study are included in the article/supplementary material, further inquiries can be directed to the corresponding author.
